# A Qualitative Study of Perspectives of Black Women on Autonomy and Motivational Interviewing

**DOI:** 10.1089/whr.2022.0094

**Published:** 2023-02-22

**Authors:** Emily F. Gregory, Peter F. Cronholm, Geminesse T. Johnson, Adya I. Maddox, Katherine Kellom, Lisa D. Levine, Scott A. Lorch, Alexander G. Fiks, Kenneth Resnicow

**Affiliations:** ^1^ Department of Pediatrics, University of Pennsylvania Perelman School of Medicine, Philadelphia, Pennsylvania, USA.; ^2^ Clinical Futures, Children's Hospital of Philadelphia, Philadelphia, Pennsylvania, USA.; ^3^ PolicyLab, Children's Hospital of Philadelphia, Philadelphia, Pennsylvania, USA.; ^4^ Department of Family Medicine and Community Health, University of Pennsylvania Perelman School of Medicine, Philadelphia, Pennsylvania, USA.; ^5^ Maternal Fetal Medicine Research Program, Department of Obstetrics and Gynecology, University of Pennsylvania Perelman School of Medicine, Philadelphia, Pennsylvania, USA.; ^6^ Department of Health Behavior and Health Education, School of Public Health, University of Michigan, Ann Arbor, Michigan, USA.

**Keywords:** adverse birth outcomes, health equity, interconception care, motivational enhancement, motivational interviewing, preconception care, preventive care, postpartum care

## Abstract

**Purpose:**

Motivational interviewing (MI) is an evidence-based strategy to modify health behaviors, including some risk factors for adverse birth outcomes. Black women, who have disproportionately high rates of adverse birth outcomes, have reported mixed preferences on MI. This study explored the acceptability of MI among Black women who are at high risk for adverse birth outcomes.

**Methods:**

We conducted qualitative interviews with women with a history of preterm birth. Participants were English-language proficient and had Medicaid-insured infants. We purposively oversampled women whose infants had medical complexity. Interviews explored experiences with health care and health behaviors after birth. The interview guide was iteratively developed to obtain specific reactions to MI by including videos demonstrating MI-consistent and MI-inconsistent counseling. Interviews were audio recorded, transcribed, and coded following an integrated approach in which we applied *a priori* codes related to MI and allowed themes to emerge from the data.

**Results:**

We interviewed 30 non-Hispanic Black women from October 2018 to July 2021. Eleven viewed the videos. Participants emphasized the importance of autonomy in decision-making and health behavior. Participants expressed a preference for MI-consistent clinical approaches, including autonomy support and building rapport, considering them respectful, nonjudgmental, and likely to support change.

**Conclusions:**

In this sample of Black women with a history of preterm birth, participants valued an MI-consistent clinical approach. Incorporating MI into clinical care may improve the experience of health care among Black women, thus serving as one strategy to promote equity in birth outcomes.

## Introduction

Birth outcomes are worsening in the United States and demonstrate persistent racial inequities.^1–3^ These trends have numerous drivers, some of which may be modifiable at the individual level.^4–7^ Clinicians require acceptable strategies to tailor health promotion for those with high risk of adverse birth outcomes, particularly Black women and those who have already experienced complications of pregnancy.^8^ For example, preterm birth is associated with modifiable health risks, which may influence future birth outcomes and long-term health.^5,8–10^

Motivational interviewing (MI) is an evidence-based behavior change strategy used in health care settings.^11^ MI has been applied during the preconception, prenatal, postnatal, and interconception periods to address risk factors for adverse birth outcomes (*e.g.*, tobacco use, weight management).^12–15^ The interconception period is an important time for health promotion after complications of pregnancy because ∼60% of US births are repeat births and complications tend to recur.^8,16^ In addition, the association between pregnancy complications and cardiovascular risk suggests benefits to sustained health promotion, even for those who do not go on to have another pregnancy.^5,9,10^

MI uses shared agenda setting, reflective listening, and eliciting “change talk” while supporting patient autonomy and enhancing motivation for change. Shared agenda setting and autonomy support are consistent with the principles of Reproductive Justice, a human-rights-based framework that centers marginalized groups to address inequities.^17,18^ While equity-oriented health frameworks often de-emphasize the role of individual behaviors, MI may be one component of individualized, equity-oriented health care.^18^

However, findings on Black women's preferences for MI are mixed.^19–21^ There are racial differences in both trust in clinicians and preferences for shared-decision making.^22–24^ One survey found that Black people were more likely than white people to prefer clinician-driven decision-making, but that women of reproductive age had stronger preferences for shared decision-making.^23^ Outcomes following MI interventions have also varied by race, with trials addressing tobacco and substance use suggesting that traditional educational approaches may better support change for Black participants than an MI approach.^22,25^ Yet, MI has been successful in interventions tailored for Black populations, including interventions to reduce problematic alcohol use and unsafe sex practices, and to improve diabetes management.^26,27^

Given the mixed findings for acceptability of MI among Black women and the potential role of MI in addressing risk factors for adverse birth outcomes, the goal of this analysis was to explore the perspectives of Black women at high risk for adverse birth outcomes on MI and the clinical approaches they experienced as helpful in supporting healthy behaviors.

## Methods

### Setting and participants

This cross-sectional qualitative study was part of a larger project on adaptation of care management strategies to address interconception health and health care access after preterm birth.

Participants were English-language proficient and had Medicaid-insured preterm infants who received primary care at sites affiliated with an academic pediatric health system. Preterm was defined as birth at <34 weeks or at 34–36 weeks with a modifiable risk (*e.g.*, tobacco use). This definition was intended to align with likely participants in the planned care management intervention, emphasizing the perspectives of those with known risk factors for adverse birth outcomes or substantial health care needs after pregnancy.

Among women meeting inclusion criteria, we purposively sampled in two phases (Table 1). First, we sampled women seeking infant care within 9 months of birth at one urban primary care site chosen for its large size and high proportion of Medicaid-insured infants. Second, we purposively sampled women whose preterm infants had medical complexity, defined as participation in infant care management provided through the pediatric health system.^28^ We expected these participants to have more to share on different clinical approaches because of their experiences with pediatric care management. We also theorized they might have distinct perspectives because having an infant with medical needs influences maternal health and well-being.^29,30^ Because those eligible for the second phase represented a smaller group, we expanded the criteria to within 3 years of birth, at three urban care sites with high proportions of Medicaid-insured infants. Potentially eligible participants were identified from pediatric electronic health records.

**Table 1.** Phases of Interviewing

	First phase	Second phase	Rationale for changes
Goals	Explore experiences with health care and health behavior after preterm birth.	Refine exploration of perspectives on care management or MI, health care, and health behavior after preterm birth.	Focus on acceptability and perceived efficacy of specific health care strategies that emerged from Phase 1 interviews as promising.
Inclusion criteria	• Preterm birth • Medicaid-insured infant • One primary care site	• Preterm birth • Medicaid-insured infant • Infant with medical complexity who receives care management • Three primary care sites	Because medical complexity is less common than preterm birth, we expanded some criteria to reach an adequate sample.
Timing	Within 9 months of birth	Within 36 months of birth	
MI video	No	Yes	Provide standardized examples rather than relying on reported characteristics of experienced health care.
Sample	17	13	Perspectives and experiences aligned across samples, allowing thematic saturation with a smaller sample.

MI, Motivational Interviewing.

This study was reviewed by the Institutional Review Board at our institution and was considered exempt.

### Interview guide and MI theory

The interview guide (Supplementary Data) initially focused on experiences with health care, health care access, and health behavior following birth. Questions were developed with reference to the Self-Determination Theory (SDT), often considered the de facto theory of MI, which focuses on individuals' needs for autonomy, competence, and relatedness.^31^ Autonomy is defined as feeling “volitional, as the originator of one's actions,” competence means feeling capable of achieving ones goals, and relatedness describes feeling connected to and understood by others.^31^

SDT also distinguishes between autonomous and controlled motivation. Autonomous motivation is driven by one's own values and goals, while controlled motivation is driven by external rewards or internalized guilt or shame. Autonomy in SDT is distinct from independence, which describes whether one relies on others' input, because one can autonomously choose to yield decision-making to others. Because autonomous motivation is associated with sustained behavior change, autonomy support is the core of MI.^31^ During MI, clinicians enhance autonomous motivation by exploring values and understanding people's lived reality to find meaning in behavior change.^32^

We iteratively developed the interview guide to explore preferences for clinical styles and strategies to support health.^33,34^ We wanted to ensure that data reflected participants' own reflections on wellness and health, and also to obtain data specific to adapting a health care intervention.^28^ For example, when we began interviewing participants experienced with pediatric care management, we asked specifically how this approach might help address participants' own needs.

We also incorporated a video demonstrating MI-consistent and MI-inconsistent approaches to counseling in the second phase of interviewing. The video was developed to train pediatricians to use MI to support children's healthy behaviors, and illustrated a doctor speaking with a mother.^35^ Both actors in the video were female. The actor portraying the mother was Black and the actor portraying the doctor was white. In both approaches, the clinician addressed the same teaching points. In the MI-inconsistent approach, the clinician was more directive and less empathetic. The clinician set the agenda, provided unsolicited advice, and did not reflect or incorporate the mother's perspective into the conversation.

In contrast, in the MI-consistent approach, the clinician asked permission to provide advice, elicited and reflected the mother's perspective, and engaged in change talk related to behavioral goals. The video was posted to a private YouTube station and a link was sent to participants allowing viewing before or during the interview. Participants viewed the MI-inconsistent approach before the MI-consistent approach.

The interview guide did not specifically ask about experiences with racism.

### Interviews

Interviews were conducted by four female researchers. Two interviewers reported their race and ethnicity as non-Hispanic white, one as non-Hispanic Black, and one as non-Hispanic biracial Asian and white. All interviewers were experienced in qualitative research and research in maternal–child health settings, including NICU care, home visiting, and newborn primary care. Interviewers had no prior relationship with participants and introduced themselves as research employees of the pediatric hospital. Interviews occurred in person or by telephone, privately between the interviewer and the participant, although in some cases, young children of participants were present. Interviews continued until we reached saturation on all themes.

Participants were asked to complete a demographic survey at the end of the interview to contextualize the sample. This survey included questions on race and ethnicity given that interpersonal and structural racism can be barriers to health care access and health.^4,7,36^ Participants were offered gift cards to offset the burdens of participating in research. Interviews were audio-recorded and professionally transcribed (ADA Transcription, Mount Holly, NJ, USA).

### Analysis

We used an integrated approach to coding.^37^ Using NVivo software (Version 12.0, 2018), we first created an *a priori* codebook reflecting constructs that informed interview guide development, for example, autonomy and health care experiences. Throughout coding, we also allowed themes and subthemes to emerge in a grounded approach to assess content not represented in our *a priori* assumptions. During initial interviews and coding, interviewers met regularly with other study team members (E.F.G. and P.F.C.) to debrief impressions and discuss code book and interview guide revisions.

Coding was completed by two nursing students, a public health student, a premedical student (G.J.), a research coordinator who also conducted interviews (A.M.), and a physician (E.F.G.). Coders had training in qualitative research and were orientated to the project and underlying theories. All transcripts were double coded and reviewed for consistency. Differences in coding were resolved through discussion and functioned as opportunities to clarify the coding scheme.^38^ To further ensure consistency across coders, one person (E.F.G.) coded at least two interviews with another coder. After coding meetings, we circulated memos related to clarifications and changes to the coding scheme.

Debriefing meetings and memos were intended to promote reflexivity (*i.e.*, examination of the extent to which researchers' experiences and beliefs may influence data collection or analysis).^39^ After starting the second phase of interviewing, we recoded interviews from the first phase to revisit assumptions and interpretations. Coders and interviewers represented various races, ethnicities, and personal experience as parents or with health care, which we considered a strength in interpretation of the data. Our consent process did not account for recontacting participants. Thus, participants did not review transcripts or provide feedback on findings.

## Results

**FIG. 1.**
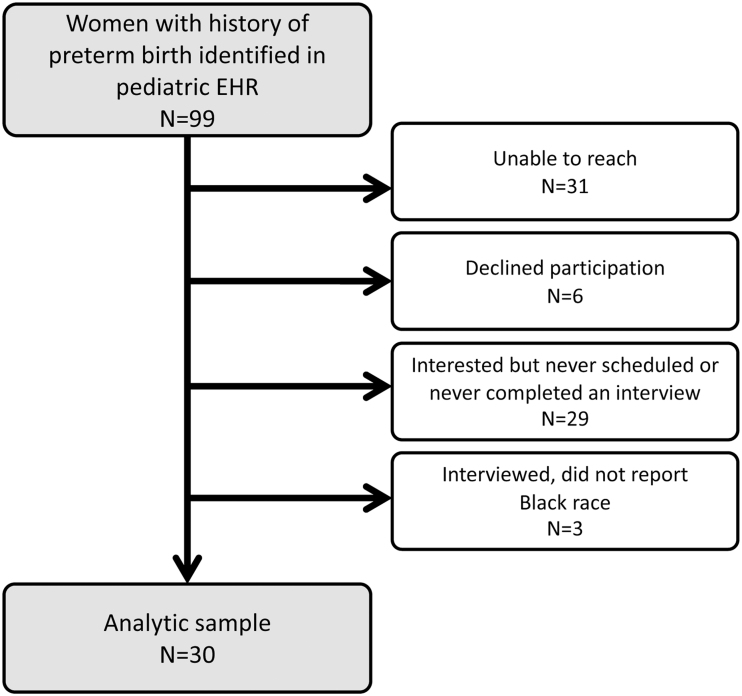
Eligible and completed interviews.

We attempted to reach 99 women who met the inclusion criteria and interviewed 33 participants. There were no inclusion criteria related to race or ethnicity, however, our focus on Medicaid-insured patients with birth complications seeking care at our institution yielded a sample that predominantly reported Black race. In this analysis, we consider findings from the 30 participants who reported non-Hispanic Black race and ethnicity (Fig. 1). Interviews occurred from October 2018 to July 2021 and lasted a mean of 36 minutes (range 18–55) (Table 2). There was race concordance during three interviews. Thirteen women participated in the second phase of interviewing, and 11 viewed the MI videos.

**Table 2.** Characteristics of Sample and Interviews

Mean (range), or no. (%)	Infant without medical complexity (first phase) (***N*** = 17)	Infant with medical complexity (second phase) (***N*** = 13)
Participant characteristics		
Participant age, years	27 (18–41)	32 (24–42)
Infant age at interview, months	4 (2–8)	19 (7–34)
Gestational age at birth, weeks	31 (25–36)	27 (22–35)
Black or African American race	17 (100%)	13 (100%)
Hispanic ethnicity	0 (0%)	0 (0%)
Educational attainment		
Less than high school	3 (18%)	2 (15%)
High school	9 (53%)	6 (46%)
Some college	4 (23%)	3 (23%)
Bachelor's or graduate degree	1 (6%)	2 (15%)
Participant relationship with infant's biological father		
Married or live together	9 (52%)	1 (8%)
Don't live together, parents in a relationship	1 (6%)	3 (23%)
Don't live together, father involved with child	6 (35%)	6 (46%)
No relationship	1 (6%)	3 (23%)
Interview characteristics		
Viewed MI video	0	13 (87%)
Timing of interviews	October 2018–February 2020	December 2020–July 2021
Interview length, minutes	39 (25–51)	34 (18–55)

Participants in the second phase of interviewing (with medically complex infants) were older than women in the first phase (mean 31 years vs. 27 years), had shorter pregnancies (mean 27 weeks vs. 31 weeks), and were further from birth at the time of the interview (mean 19 months vs. 4 months). There was convergence on themes from participants across both phases of interviewing.

Among participants who viewed the videos, they universally preferred the MI-consistent style to the MI-inconsistent style. They described the MI-consistent style as more respectful and less judgmental, and more likely to lead them to adopt positive health behaviors, compared with the MI-inconsistent style. In this study, we describe themes related to preferred clinical styles, grouped by SDT constructs of autonomy, relatedness, and competence (Table 3). Because of the nature of our research question, we focus on themes related to health care; however, participants also discussed aspects of health promotion unrelated to health care.

**Table 3.** Summary of Themes

Autonomy and autonomy support • Autonomy and independence are valued and linked to normative expectations around parenting • Receipt of MI-consistent autonomy supportive care varied across different postpartum health topics • Participants valued MI-consistent autonomy supportive care • MI-consistent autonomy support contributes to motivation
Relatedness • MI-consistent care builds rapport by demonstrating respect and nonjudgment • Clinical teams bear the responsibility for building rapport with all patients • Rapport with clinical team contributes to motivation
Competence • Knowledge deficits sometimes contribute to lower perceived competence

### Autonomy and autonomy support

Participants consistently valued autonomy and independence and linked these attributes to normative expectations around parenting roles. In response to a question about whose advice she listened to, one participant emphasized the importance of her autonomy, stating, “Mine. I mean, I get advice from everybody, but at the end of the day, I listen to mine. I do what's best for me.” Linking autonomy and independence to parenting roles, another participant reported “I don't like to overwhelm anybody else with my problems… I'm more so like independent when it comes to myself and [my baby].” Another participant told us “[having a baby] was my choice so I guess [my priorities] was more so of a mother instinct. It just came.”

In reflecting on past clinical experiences, participants reported varying levels of autonomy support across different health topics. Low autonomy support was particularly noted around contraception, where participants sometimes felt their preferences and expertise about their own bodies were questioned.

For example, one participant reported that when she told her doctors she wanted a tubal ligation “They wasn't happy at first… They were saying basically that's a permanent thing… if you're on birth control you could always stop it and be pregnant again, but I already knew what I wanted.” Sometimes low autonomy support was related to unmet contraceptive needs, as in the case of a participant who said, “[The contraception my doctor recommended would] go inside and I really don't want to do something like that. I'd rather just take the pill and she was saying that now that I have a baby I might forget to take it so she was giving me those options and I really don't want those options.”

Autonomy support was similarly lacking in advice about self-care and sleep, which did not account for participants lived reality. As one participant reflected, “they told me to rest and to get other people to try to help me so I can heal properly, but it was really, really hard to do that because his dad is always at work. He wanted to do more hours since we have a newborn baby. And I needed to get to the grocery store. I needed to cook for the kids. I needed to clean. It was the baby's doctor, my doctors… a lot of people were saying the same thing, but it's like, I couldn't.” Another participant told us, “They always say, ‘sleep when the baby sleeping’… it's helpful [advice], but it's stupid, because if I'm sleeping every time the baby's sleeping… you won't get nothing done.”

In contrast, participants generally described higher levels of autonomy support around infant feeding decisions. For example, one told us, “I decided [to breastfeed] before I had her… [and I breastfed] for like three weeks. [Then] it was too much… I told [my doctors] that I didn't want to do it no more. They told me that I don't have to.”

In commenting on past clinical experiences, participants typically described a preference for autonomy supportive care, particularly health care that respected their own expertise. Discussing her child's pediatrician, one participant said, “Understanding that premature children do have some problems, but when you know your own child, sometimes you feel like someone can't really tell you what the problem is when you already know. So I have had a problem with some doctors feeling like their answer is the best answer and no one else's answer.” Another participant described her new primary care doctor saying “I really like her… I felt like she heard me… really trying her best to understand what my needs are and trying to help me with that.”

Participants who viewed the videos noted that the MI-consistent approach supported autonomy while also respectfully providing the needed recommendations. After watching the video one participant reflected, “For me, personally, I'm not getting the communication I need from my primary [care doctor]… certain things, I felt like need to be addressed more. As far as, if you know I'm smoking cigarettes and tell me stop smoking the cigarettes. Nobody can tell you what to do, but they can definitely give a suggestion, right?”

Another participant specifically noted that the MI-consistent approach in the video might enhance her autonomous motivation, stating “when you try to listen to me and make me participate in whatever decision that is being made, it makes me feel more committed to it, to change. Because if I'm kind of making the decision, I'd like to see it through. It makes me feel empowered.”

### Relatedness and building rapport

Participants noted that MI-consistent approaches improved their clinical experiences by enhancing relatedness and building rapport. In both the video and their own clinical experiences, MI-consistent approaches were viewed as less judgmental. Commenting on the video, one participant said “the [MI-inconsistent style] would be okay for some people, but then for the next person, it might not be because some people feel like they're being judged.” Another participant, when asked which approach was more similar to her experiences with her own doctors, said “I feel like it's a mixture, but more so the [MI-inconsistent] approach… when she's talking to me, I'm kind of having my own conversation in my head. And I'm trying not to take it too hard or feel like I'm being judged.”

A third participant also noted that communication can influence patient experience and further emphasized that ensuring high-quality communication is the responsibility of the health care team, saying “Sometimes doctors do have to go over crash courses to see how they can better communicate… I felt like [the doctor in MI-inconsistent video] was out of line as well because you don't really say things like that.”

Participants also noted that clinicians need to be responsive to patients' emotional state to build rapport and improve clinical experiences. One woman described an ultrasound where she was informed her fetus might have difficulty growing because of fibroids. “I was really, really devastated by that… I was asking [the doctor], well, is there anything I can do, is there anything I need to change, should I eat a certain way, should I rest more? And he was literally just like, you just have to wait and see…. It was literally just very cold.”

One participant identified building rapport as a strategy to support motivation. After watching the videos, she commented, “Listen, it's like if you talk to somebody real nice and like explain to them in a way, in a nice way then—and make them understand, maybe they'll be like, okay, okay—or maybe I would listen to what you're saying and try what you're talking about.”

### Competence

Participants rarely described competence or knowledge deficits as barriers to health care access or health behaviors. However, they did sometimes want specific, individualized information to support competence, suggesting that low knowledge may be linked to lower perceived competence. This was noted in the quote above where a participant requested, but did not receive, detailed information about mitigating problems associated with fibroids. In another example, a participant explained that she was not taking prenatal vitamins saying, “They say take the vitamins, but they never really say what's a good vitamin. You could be in the pharmacy… and there's a billion vitamins… It's like which one do they recommend?… I don't want to walk in the store and just be picking up anything.”

## Discussion

In this sample of Black women who experienced preterm birth, participants valued clinical approaches that supported autonomy by considering their lived reality, values, emotions, and expertise. Participants consistently described MI-consistent clinical approaches as acceptable or preferred. They reported that autonomy was an important contributor to their decision-making. These findings suggest that MI-consistent care is acceptable to Black women in the prenatal, postnatal, and interconception periods.

Our findings are consistent with the principles of Reproductive Justice, a framework that recommends structural changes to locations and workforce required for prenatal and birth care, as well as restructuring individual health care encounters.^17,40,41^ Our findings apply to this later context and suggest that strategies such as MI and shared decision-making, which are already familiar to health care systems, may be well-received individual-level strategies. These findings are also consistent with recommendations from the National Academy of Medicine to address health equity through implementation of patient-centered models of care.^42^

Our findings contrast with some prior reports that Black women may prefer a more directive communication style.^19,20^ Participants in our study were slightly younger at the time they were interviewed, so our contrasting findings may agree with reports that age is associated with preferences for shared decision-making.^23,43,44^ In addition, our study differed from prior work in that participants were primed to consider their role as parents as they reflected on their experiences with health care and health behavior. In our sample, participants linked autonomy and independence to their role as parents, so this study frame may have made MI seem particularly attractive. Prior work on preferences about communication styles has, similar to our study, been cross-sectional.

Inconsistencies across different studies may reflect cohort effects, or other differences between different populations, or may suggest that preferences change across the life course or in different health care contexts. Either way, accepting variation in preferences is an important consideration for efforts to improve communication in health care.

Our exploration of MI relied, in part, on one video. Participants may have responded to something in this video separate from the intended distinction. For example, the portion with the MI-consistent approach was longer than the MI-inconsistent portion and participants always viewed the MI-inconsistent version first. In addition, the video demonstrated parent encounters on behalf of a child rather than adults receiving care for themselves. However, participants readily connected the video to their own health care experiences. Moreover, responses to the video were consistent with what participants told us about their values and health care experiences throughout the interviews. It is important to note that participants did not actually undergo MI and may have expressed distinct preferences following participation in an MI intervention.

This study is subject to several other limitations. First, our sample was limited to Black women with Medicaid-insured preterm infants living in an urban area and seeking infant care at a tertiary care pediatric health system. Findings may not be broadly generalizable, even to others who share some characteristics with our sample, for example, Black race. In particular, findings may not be generalizable to those who do not regularly seek care, live in nonurban areas, are distrustful of health care-related research, or who had uncomplicated pregnancies. In addition, as interviews were conducted by health system-affiliated staff, participants may not have fully expressed criticisms of care.^39^ There was not racial concordance between most interviewers and participants. Although interviews occurred by phone, which may have made concordance less salient, this may have influenced responses.^39^

## Conclusions

This sample of Black women with a history of preterm birth expressed a strong preference for health care characterized by autonomy support and relatedness and thought these characteristics of clinical care could drive positive health behavior. Participants described mixed experiences with autonomy support in their past clinical experiences. Better incorporating MI throughout reproductive care for Black women is acceptable and may improve the experience of care for women at high risk for adverse birth outcomes.

## Supplementary Material

Supplemental data

## Abbreviations Used

MI: motivational interviewing
SDT: Self-Determination Theory
