# Supplementary material for: A Qualitative Study of Perspectives of Black Women on Autonomy and Motivational Interviewing
Source: Womens Health Rep (New Rochelle). 2023 Feb 22;4(1):94–102. doi: 10.1089/whr.2022.0094 (PMC9983129; doi:10.1089/whr.2022.0094)
Supplement: Supplemental data [file Suppl_Data.docx]

1. Tell me about how you are taking care of yourself since your baby was born.
2. After your baby was born, what did your doctors and nurses tell you in terms of taking care of yourself?
3. Tell me about your priorities for yourself and your family around the time you brought [BABY NAME] home from the hospital.
4. Was there a time during or after your pregnancy when your health care team wanted you to make a change? What were those conversations like?
5. I’m going to ask you to watch a video clip that show two different styles doctors and nurses sometimes use when they’re talking with people about making changes and then I’m going to ask you some questions about those styles of talking with people.
   1. How do you feel about the way the doctor talked to the mom in the two clips?
   2. How do your doctors and nurses talk with you compared to the doctor in the video?
   3. What feels helpful as far as how people talk to you when you are trying to make changes in your life?
   4. How would you feel about having someone from [CARE MANAGER NAME]’s team talk with you about making changes for your own health like this?
6. If someone from [CARE MANAGER NAME]’s team was trying to support you, or people in similar situations, *after* pregnancy…
   1. What would you think of that?
   2. What would be the most important things to address?
   3. Would you be comfortable with someone from [CARE MANAGER NAME]’s team communicating with your doctors and nurses directly?
   4. How would you want to connect with this person, and how often?
